# The influence of concomitant proton pump inhibitors use on treatment efficacy in hepatocellular carcinoma patients receiving immune checkpoint inhibitors: a systematic review and meta-analysis

**DOI:** 10.3389/fimmu.2026.1717420

**Published:** 2026-02-03

**Authors:** Yangfei Duan, Qitai Zhao, Shumin Feng, Wei Jing, Dan Wang, Yi Zhang

**Affiliations:** 1 School of Public Health, Zhengzhou University, Zhengzhou, Henan, China; 2 Biotherapy Center and Cancer Center, The First Affiliated Hospital of Zhengzhou University, Zhengzhou, Henan, China; 3 School of Life Sciences, Zhengzhou University, Zhengzhou, Henan, China; 4 State Key Laboratory of Metabolic Dysregulation & Prevention and Treatment of Esophageal Cancer, Tianjian Laboratory of Advanced Biomedical Sciences, Academy of Medical Sciences, Zhengzhou University, Zhengzhou, Henan, China; 5 Zhongyuan Cell Therapy and Immunotherapy Laboratory, Henan Academy of Innovations in Medical Science, Zhengzhou, Henan, China

**Keywords:** hepatocellular carcinoma, immune checkpoint inhibitors, overall survival, progression-free survival, proton pump inhibitors

## Abstract

**Background:**

Despite the significant survival benefit offered by immune checkpoint inhibitors (ICIs) in patients with hepatocellular carcinoma (HCC), a subset of patients still develop drug resistance. Recent evidence suggests that proton pump inhibitors (PPIs) may influence the therapeutic efficacy of ICIs, but the clinical relevance of this interaction remains unclear. This meta-analysis aims to systematically evaluate the association between concomitant PPIs use and clinical outcomes in HCC patients receiving ICIs therapy.

**Methods:**

A comprehensive search of the PubMed, Embase (via Ovid), and Web of Science databases was conducted to identify relevant studies published before May 14, 2025. The primary endpoints of the meta-analysis were overall survival (OS) and progression-free survival (PFS).

**Results:**

Five retrospective studies comprising a total of 1,257 patients were included, of whom 606 (48.2%) received PPIs concurrently with ICIs. The meta-analysis showed no statistically significant association between PPIs use and either OS (HR: 1.04, 95% CI: 0.88-1.24, P = 0.64) or PFS (HR: 0.92, 95% CI: 0.74-1.14, P = 0.44). These findings were further supported by a Bayesian sensitivity analysis performed to address the uncertainty inherent in a limited number of studies.

**Conclusion:**

Based on the current evidence from retrospective studies, concomitant use of PPIs does not appear to significantly affect survival outcomes in HCC patients treated with ICIs. However, given the inherent limitations of the included studies, this conclusion should be interpreted with caution and warrants validation through prospective investigations.

**Systematic review registration:**

https://www.crd.york.ac.uk/PROSPERO/view/CRD420251026028, CRD420251026028.

## Introduction

Hepatocellular carcinoma (HCC) represents a major global health problem and is the third most common cause of cancer-related death worldwide (1). With the emergence of immunotherapy, the combination of immune checkpoint inhibitors (ICIs) and antiangiogenic agents has become the first-line therapeutic regimen for HCC. Compared with conventional treatment approaches, this combination strategy has demonstrated significantly improved therapeutic efficacy (2). The therapeutic effect of ICIs is primarily attributed to their ability to disrupt key immune checkpoint pathways, such as PD-1/PD-L1. By inhibiting the binding of these checkpoint molecules, ICIs release the ‘brakes’ on the immune system, rescuing T cells from functional exhaustion and allowing them to mount an effective antitumor response (3, 4). However, patients who do not respond to ICIs treatment may experience tumor progression and potentially suffer from significant side effects (5, 6). Therefore, optimizing treatment outcomes in HCC depends on recognizing key modifiers of ICIs efficacy (7).

In clinical practice, cancer patients receiving ICIs often require concurrent administration of other medications to manage various comorbidities—such as antibiotics, proton pump inhibitors (PPIs), and opioids (8). Different types of these concomitant medications may exert distinct impacts on the therapeutic efficacy of ICIs. Among these agents, emerging clinical evidence suggests that PPIs can modulate the gut microbiota, regulate the cellular acid-base environment, and influence inflammatory factors (9–11). Through these mechanisms, PPIs may further alter the immune response, thereby affecting the therapeutic outcomes of ICIs-based treatment. Current meta-analyses demonstrate that concomitant use of PPIs significantly diminishes the therapeutic effectiveness of ICIs among individuals diagnosed with non-small cell lung cancer (NSCLC) and urothelial carcinoma (UC) (12, 13). However, comprehensive and systematic meta-analyses investigating whether PPIs affect the therapeutic efficacy of ICIs in the treatment of HCC are currently lacking.

In this study, we conducted a meta-analysis to elucidate the association between PPIs exposure and treatment outcomes in HCC patients receiving ICIs therapy, with the aim of providing evidence for clinical medication selection.

## Materials and methods

### Search strategy

This meta-analysis adhered to the updated Preferred Reporting Items for Systematic Reviews and Meta-Analyses (PRISMA) guidelines (14), with a pre-registered protocol (PROSPERO ID: CRD420251026028). We systematically searched PubMed, Ovid (Embase), and Web of Science from inception through May 14, 2025, without language restrictions. The search strategy combined MeSH terms and free-text synonyms for three key concepts: “proton pump inhibitors”, “immune checkpoint inhibitors” and “hepatocellular carcinoma”. Complete search syntax for each database is provided in **Supplementary Table S1**.

### Study selection criteria

We kept studies that (1): Enrolled people with HCC treated with ICIs; (2) Gave hazard ratios (HRs) and 95% confidence intervals (CIs) for overall survival (OS) or progression-free survival (PFS), comparing patients who took PPIs with those who did not. We dropped single-case reports, reviews, pooled analyses, commentaries, animal work, and papers with missing data. When the same patients appeared in more than one article, we used the newest and most complete one.

### Data extraction protocol

Two reviewers worked alone, filling out the same form: first author, year, design, country, sample size, ICIs regimen, PPIs use, and multivariable HRs (95% CIs) for OS or PFS. If numbers were missing, we emailed the authors or checked supplements. Every entry was double-checked before locking the file.

### Quality assessment methodology

The Newcastle-Ottawa Scale (NOS) was used to assess the quality of the included retrospective studies (15). Evaluations were conducted across three dimensions: selection of study participants, comparability between groups, and outcome measurement. Studies with a total score of 9 were considered methodologically rigorous, while those scoring ≥7 were regarded as high-quality. Two researchers performed the assessments independently, and any discrepancies were resolved through discussion.

### Statistical methods

All statistical analyses were conducted using Review Manager (RevMan) 5.4.1 and Stata SE 15.0. Effect sizes are reported as HRs with corresponding 95% CIs. Given the limited number of included studies (n=5) and anticipated clinical heterogeneity, the primary meta-analysis under the frequentist framework employed a more conservative random-effects model. All hypothesis tests were two-sided, with a P-value < 0.05 considered statistically significant.

To address the increased estimation uncertainty inherent in meta-analyses with few studies, a supplementary Bayesian random-effects model was also applied. The model was implemented using the ‘bayesmeta’ package in R software (version 4.5.1), with a non-informative prior specified for the overall effect size (log HR) and a uniform distribution used as a weakly informative prior for the between-study heterogeneity parameter (τ). To evaluate the robustness of the results, a sensitivity analysis employing Jeffreys prior was additionally performed.

Potential biases were assessed by visual inspection of funnel plots together with Begg’s and Egger’s tests for publication bias. Sensitivity analysis was carried out by sequentially excluding individual studies to examine the stability of the pooled estimates.

## Results

### Search results and study selection

**Figure 1:**
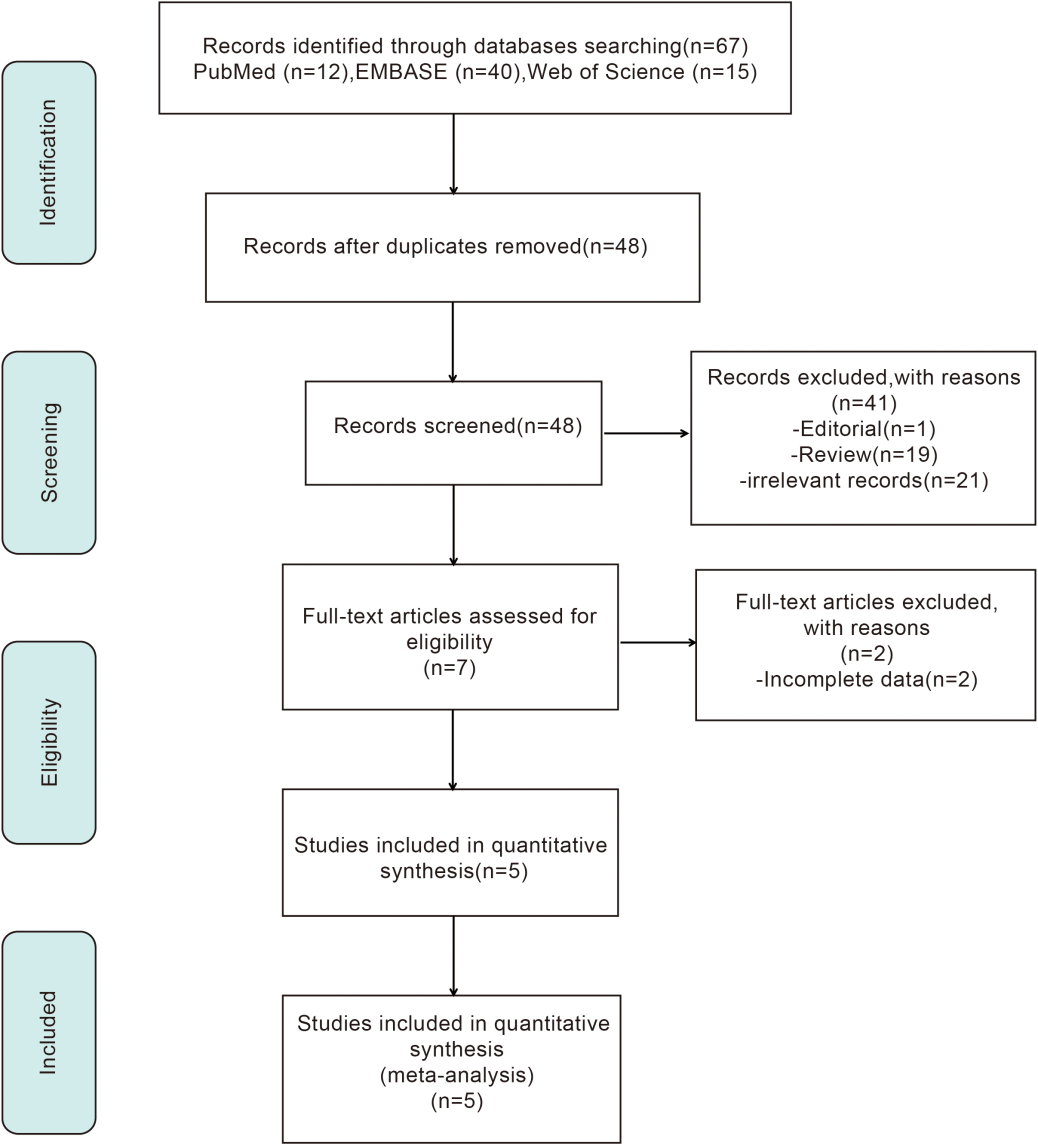
Flow diagram of the study screening and selection process.

A database search retrieved 67 articles. After initial screening, seven articles proceeded to full-text evaluation. Following assessment, two studies were excluded due to incomplete data (16, 17), and five studies met the criteria and were included for pooled analysis (18–22). The PRISMA flow diagram illustrates the selection process (**Figure 1**).

As detailed in **Table 1**, the relevant information of the five included studies is presented. From these, we note that each study was retrospective, and all five reported OS data, while only three reported PFS data. The studies by Jun et al. (20) and Hobeika et al. (19) utilized univariate Cox regression models, whereas Ng et al. (21) employed a multivariate Cox regression model with covariates including Eastern Cooperative Oncology Group Performance Status (ECOG score), Child-Pugh grade, HCC etiology, and Alpha-Fetoprotein (AFP), among others. Furthermore, Wang et al. (22) and Hatanaka et al. (18) applied propensity score matching (PSM) and inverse probability of treatment weighting (IPTW), respectively, to control for confounding factors. In terms of conclusions, each study indicated that PPIs use was not associated with patient survival outcomes. However, a subgroup analysis in one of the studies found that PPIs use was associated with an increased risk of death in patients with high HBV DNA levels. Finally, three studies were rated as high-quality (scores of 7-8), and two studies as medium-quality (score of 6).

**Table 1:** Baseline demographic and clinical profiles of the five included studies.

Author	Year	Study design	Study period	Country/region	PPIs group (n)	Non-PPIs group (n)	ICIs regimen	Outcomes	Statistical analysis (cox regression model)	Adjusted covariates	NOS score
Hobeika et al. (19)	2024	Retrospective	–	Germany	86	66	PD-L1	OS, PFS, AEs	Univariate	None	6
Jun et al. (20)	2021	Retrospective	2017-2019	Europe,United States,Asia	92	221	PD-1/ CTLA-4	OS, ORR, DCR, AEs	Univariate	None	6
Ng et al. (21)	2024	Retrospective	2015-2019	Singapore	101	67	PD-1/PD-L1/CTLA-4	OS, PFS, ORR, DCR	Multivariate	ECOG, Child-Pugh, HCC etiology, and AFP levels et al.	7
Wang et al. (17)	2024	Retrospective	2020-2022	China	88	95	PD-1	OS, AEs	Multivariate (after PSM)	Age, Sex, History of alcoholism, Child-Pugh, BCLC, ALB, and Treatment et al.	8
Hatanaka et al. (18)	2023	Retrospective	2020-2022	Japanese	239	202	Atez/Bev	OS, PFS	Multivariate (with IPTW)	Age, Sex, Viral infection, BCLC stage, Child-Pugh score, ALBI score, Treatment settings, Serum albumin, Total bilirubin, and Fibrosis-4 et al.	8

ICIs, Immune checkpoint inhibitors; PPIs, Proton pump inhibitors; HCC, Hepatocellular carcinoma; PD-1, programmed cell death 1; PD-L1, programmed cell death ligand 1; CTLA-4, cytotoxic T-lymphocyte-associated protein 4; Atez, Atezolizumab; Bev, Bevacizumab; OS, overall survival; PFS, progression-free survival; ORR, objective response rate; DCR, disease control rate; AEs, Adverse Events; PSM, Propensity Score Matching; IPTW, Inverse Probability of Treatment Weighting; AFP, Alpha fetoprotein; BCLC, Barcelona Clinic Liver Cancer; ECOG, Eastern Cooperative Oncology Group Performance Status; ALB, Serum Albumin; ALBI, Albumin-Bilirubin Score; NOS, Newcastle-Ottawa Scale.

### Meta-analysis of OS

**Figure 2:**
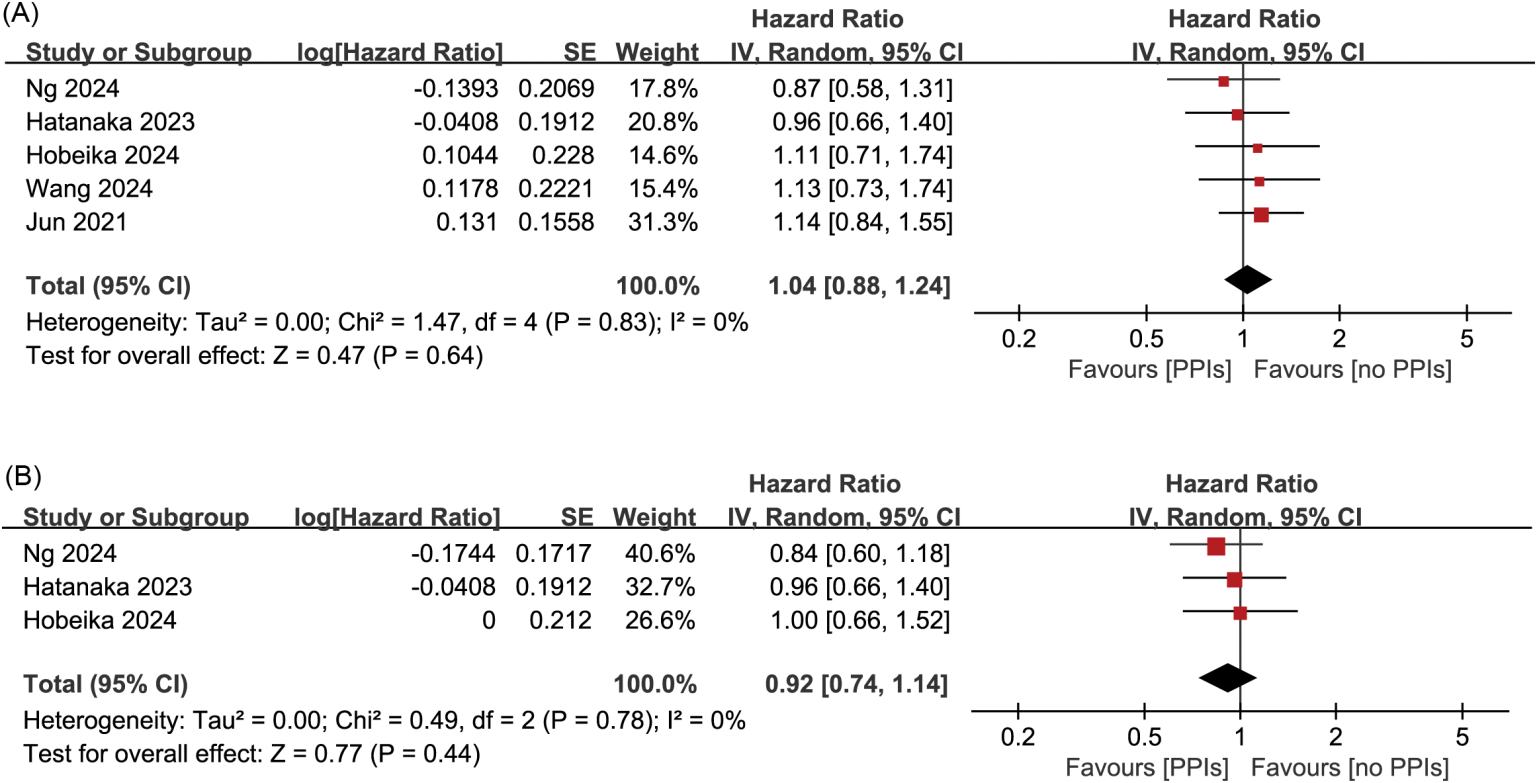
Forest plots of the meta-analysis evaluating the influence of concomitant use of PPIs on **(A)** overall survival and **(B)** progression-free survival in HCC patients receiving ICIs. PPIs, proton pump inhibitors; HCC, hepatocellular carcinoma; ICIs, immune checkpoint inhibitors.

**Figure 3:**
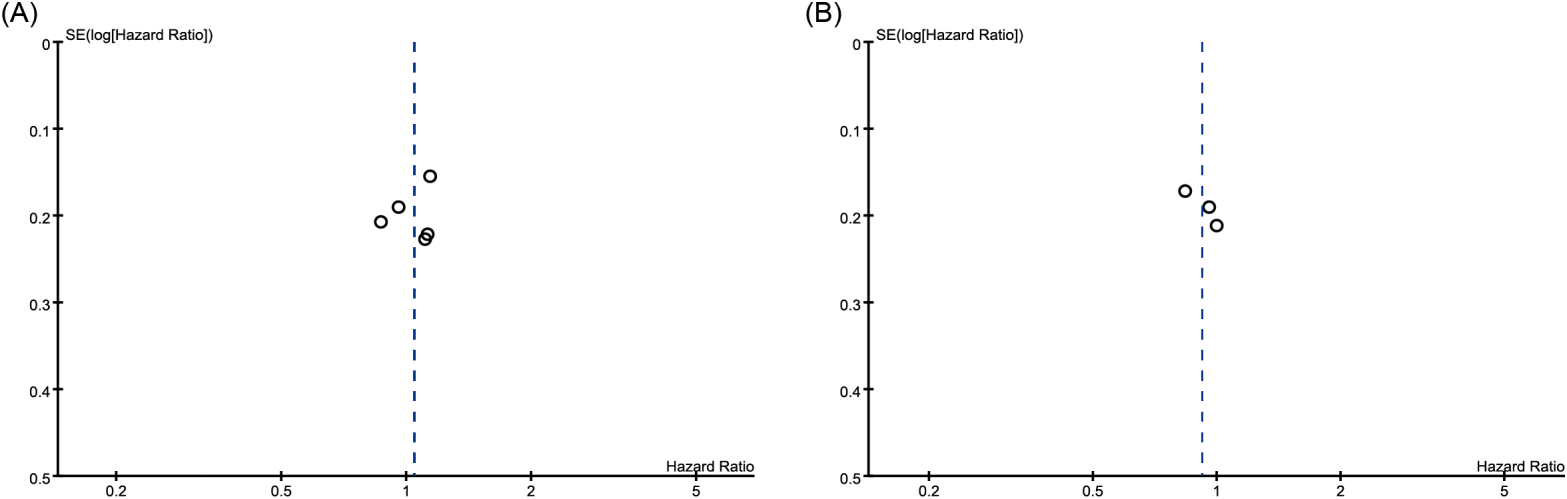
Funnel plots from the meta-analysis assessing the influence of concomitant use of PPIs on **(A)** overall survival and **(B)** progression-free survival in patients with HCC treated with ICIs. PPIs, proton pump inhibitors; HCC, hepatocellular carcinoma; ICIs, immune checkpoint inhibitors.

**Figure 4:**
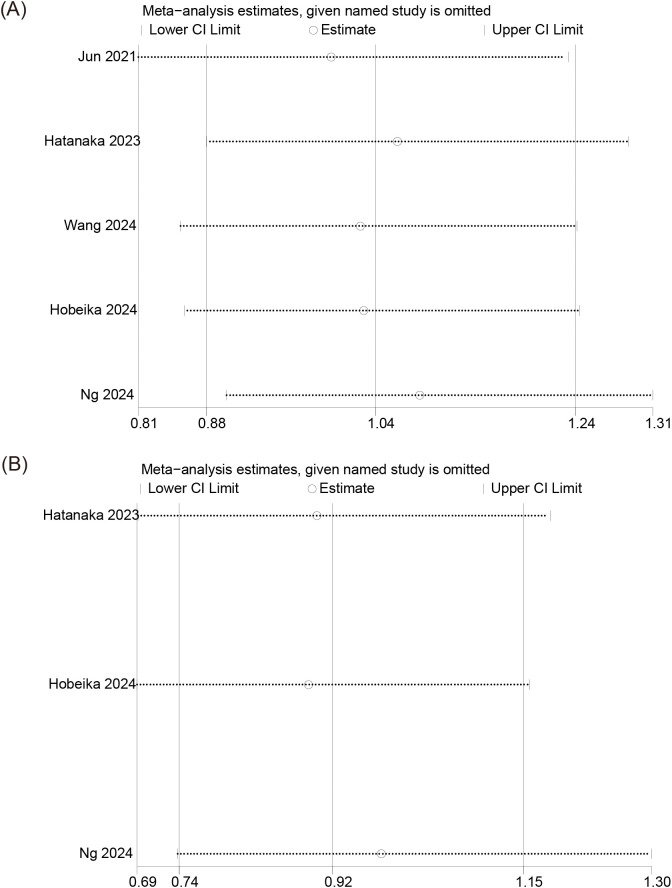
Sensitivity analysis of the influence of concomitant use of PPIs on **(A)** overall survival and **(B)** progression-free survival in patients with HCC treated with ICIs. PPIs, proton pump inhibitors; HCC, hepatocellular carcinoma; ICIs, immune checkpoint inhibitors.

The pooled analysis of OS showed a Cochran’s Q test p=0.83 and I²=0%. However, this result does not indicate the absence of heterogeneity among the five studies—it is likely due to the small number of studies included. Furthermore, given that retrospective studies are prone to clinical and methodological heterogeneity, we adopted a more conservative random-effects model. The forest plot revealed no significant association between PPIs use and patient OS (pooled HR: 1.04; 95% CI: 0.88-1.24; P = 0.64) (**Figure 2A**). In addition, no publication bias was detected based on the funnel plot (**Figure 3A**), Begg’s test (P = 0.806), or Egger’s test (P = 0.656), and sensitivity analysis confirmed the stability of the results (**Figure 4A**). Nevertheless, all findings mentioned above are constrained by the limited number of studies and should be interpreted with caution.

### Meta-analysis of PFS

Similarly, the pooled analysis for PFS was also conducted using a random-effects model. The results showed no significant association between PPIs use and PFS (pooled HR: 0.92; 95% CI: 0.74-1.14; P = 0.44) (**Figure 2B**). The funnel plot, Begg’s test (P = 0.296), and Egger’s test (P = 0.170) all indicated no publication bias (**Figure 3B**). Sensitivity analysis further supported these findings (**Figure 4B**). However, given the limited number of studies, the above results should be interpreted with caution.

### Bayesian meta-analysis of OS and PFS

**Figure 5:**
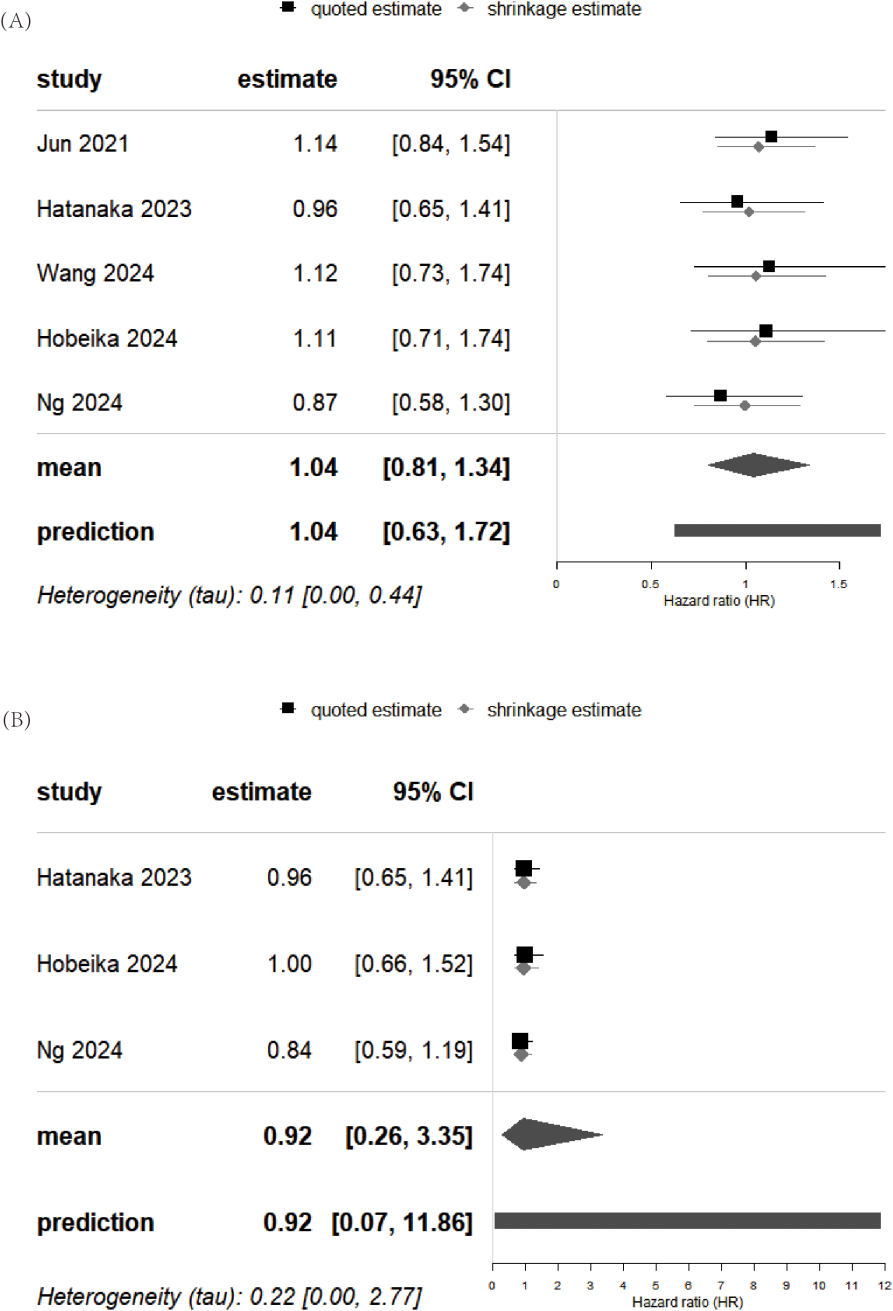
Bayesian meta-analysis of the effect of concomitant PPIs use on **(A)** overall survival and **(B)** progression-free survival in HCC patients treated with ICIs. PPIs, proton pump inhibitors; HCC, hepatocellular carcinoma; ICIs, immune checkpoint inhibitors.

Bayesian meta-analysis results indicate that PPIs also had no significant effect on either OS or PFS (**Figure 5**). However, it can be observed that the 95% credible interval (CrI) and 95% predictive interval (PrI) for PFS are considerably wider than those for OS, reflecting greater uncertainty in the PFS results (**Figure 5**). Therefore, the interpretation of these findings should be approached with additional caution.

## Discussion

In advanced cancer patients undergoing ICIs therapy, the concurrent use of multiple medications for managing comorbidities, supportive care, or dietary supplementation is prevalent, leading to a common scenario of polypharmacy (23, 24). This multi-drug regimen, however, elevates the risk of drug-drug interactions, which may interfere with ICIs efficacy through potential mechanisms such as altering gut microbiota, affecting the immune microenvironment, or disrupting pharmacokinetics (25). Among concomitant medications, PPIs-widely utilized for acid suppression-have drawn sustained attention and remain a key focus in this field of investigation.

ICIs-based therapy has emerged as an initial management strategy for individuals diagnosed with HCC (26). However, the response to ICIs varies among cancer patients, with some patients prone to developing resistance. Investigating the factors influencing ICIs efficacy is the focus of this study. Our study is the first to systematically explore the impact of PPIs on ICIs therapy in patients with HCC through a meta-analysis, thereby providing certain reference value for clinical practice.

Our pooled analysis demonstrates no statistically significant association between PPIs exposure and survival outcomes among HCC patients receiving ICIs. This contrasts with prior meta-analyses such as Chen et al. (27), which suggested detrimental effects of PPIs on ICIs survival outcomes across advanced cancers. Proposed mechanisms for PPIs-mediated attenuation of ICIs efficacy are multifaceted, potentially involving immunomodulatory effects such as downregulation of immune cell adhesion molecules and alterations in proinflammatory cytokine profiles (28). Additionally, PPIs may compromise ICIs efficacy by reducing gut microbiota diversity, a factor positively correlated with treatment response (29).

Our findings suggest that the impact of PPIs on the efficacy of ICIs may differ across tumor types. The lack of an adverse effect of PPIs on ICIs efficacy in HCC may be attributed to two factors. Firstly, this may be attributable to the unique pathophysiology of HCC: patients frequently present with underlying chronic liver disease (e.g., cirrhosis, viral hepatitis), conditions inherently associated with gut dysbiosis and impaired intestinal barrier function (30). Within this context, the incremental impact of PPIs on the microbiome may be overshadowed by pre-existing dysbiosis, diminishing observable clinical effects. Furthermore, the highly heterogeneous HCC immune microenvironment-characterized by complex interactions among diverse immune cells (T cells, macrophages) and cytokines (TGF-β,IL-6) (31)-may render it less susceptible to the relatively modest immunomodulatory effects of PPIs. Critically, PPIs may lack the capacity to effectively counteract HCC-specific immunosuppressive networks.

It is noteworthy that a primary limitation of this research lies in its exclusive inclusion of retrospective observational studies. The clinical indications for PPIs administration, such as gastroesophageal reflux disease or stress ulcer prophylaxis, may introduce systematic disparities between users and non-users with regard to baseline comorbidities, functional status, and disease severity, resulting in substantial confounding bias. Although some original studies employed multivariate Cox regression for adjustment, the covariates adjusted for were inconsistent across studies, and most did not adopt more advanced causal inference methodologies, such as propensity score matching. Consequently, the pooled HRs from this meta-analysis should be interpreted with caution when inferring a causal relationship between PPIs use and survival outcomes. The findings are better regarded as a significant associative signal, warranting further validation through rigorously designed prospective studies.

Another significant limitation of this study is the inclusion of only five studies, which to some extent compromises the reliability of conventional meta-analytical tools (such as heterogeneity tests). Specifically, the anticipated clinical and methodological heterogeneity across retrospective studies may act as an important confounding factor. It should be particularly noted that with a limited number of included studies, the statistical power of the I²statistic and Cochran’ s Q test is substantially reduced; hence, a non-significant p-value should not be regarded as strong evidence for homogeneity across studies (32). As emphasized by Zhou et al. (33), meta-analyses based on a small number of studies should refrain from drawing definitive conclusions and are advised to adopt more robust approaches–such as Bayesian models-to better account for uncertainty. Accordingly, a Bayesian meta-analysis was performed in the present study. The results indicated that, compared with OS, PFS outcomes exhibited greater uncertainty, as reflected by wider credible intervals. This finding further reinforces the need for a cautious interpretation of the current conclusions. Additionally, although publication-bias assessments and sensitivity analyses were conducted, the limited number of included studies may introduce bias in the interpretation of these auxiliary analyses. Therefore, the results of these assessments should primarily be considered descriptive references and interpreted with due caution.

This study still has certain limitations (1): The databases involved in this study are not comprehensive enough and do not include databases such as Scopus and Cochrane Library, which may lead to omissions in the literature (2). There are gaps in the information regarding PPIs exposure, preventing a more in-depth analysis. (3) Furthermore, excluding studies with missing HRs for OS or PFS may introduce selection bias. (4) Finally, the quality of the included studies requires further improvement. In conclusion, the interpretation of the results should be approached with greater caution. In the future, conducting large-scale prospective studies could help control the influence of confounding factors and provide evidence to clarify the role of PPIs in the treatment of HCC with ICIs.

## Conclusion

This meta-analysis indicates that exposure to PPIs was not found to be significantly associated with OS or PFS in HCC patients receiving ICIs therapy. However, given that the included studies were predominantly retrospective in design and residual confounding cannot be excluded, these findings should be interpreted with caution. Further investigation through well-designed prospective studies or dedicated subgroup analyses of randomized controlled trials is warranted to establish a definitive causal relationship.

## Data Availability

The original contributions presented in the study are included in the article/**Supplementary Material**. Further inquiries can be directed to the corresponding author.
