# Supplementary material for: The influence of concomitant proton pump inhibitors use on treatment efficacy in hepatocellular carcinoma patients receiving immune checkpoint inhibitors: a systematic review and meta-analysis
Source: Front Immunol. 2026 Feb 3;17:1717420. doi: 10.3389/fimmu.2026.1717420 (PMC12960652; doi:10.3389/fimmu.2026.1717420)

Pubmed(12)

((("Carcinoma, Hepatocellular"[Mesh]) OR ((((((((((((((((((Carcinomas, Hepatocellular) OR (Hepatocellular Carcinomas)) OR (Hepatocellular Carcinoma)) OR (Hepatoma)) OR (Hepatomas)) OR (Liver Cancer, Adult)) OR (Adult Liver Cancer)) OR (Adult Liver Cancers)) OR (Cancer, Adult Liver)) OR (Cancers, Adult Liver)) OR (Liver Cancers, Adult)) OR (Liver Cell Carcinoma)) OR (Carcinoma, Liver Cell)) OR (Carcinomas, Liver Cell)) OR (Cell Carcinoma, Liver)) OR (Cell Carcinomas, Liver)) OR (Liver Cell Carcinomas)) OR (Liver Cell Carcinoma, Adult))) AND (("Proton Pump Inhibitors"[Mesh]) OR ((((Inhibitors, Proton Pump) OR (Proton Pump Inhibitor)) OR (Inhibitor, Proton Pump)) OR (Pump Inhibitor, Proton)))) AND (("Immune Checkpoint Inhibitors"[Mesh]) OR ((((((((((((((((((((((((((((((((((((((((((((((Checkpoint Inhibitors, Immune) OR (Immune Checkpoint Blockers)) OR (Checkpoint Blockers, Immune)) OR (Immune Checkpoint Inhibitor)) OR (Checkpoint Inhibitor, Immune)) OR (CTLA-4 Inhibitors)) OR (CTLA 4 Inhibitors)) OR (Cytotoxic T-Lymphocyte-Associated Protein 4 Inhibitors)) OR (Cytotoxic T Lymphocyte Associated Protein 4 Inhibitors)) OR (Cytotoxic T-Lymphocyte-Associated Protein 4 Inhibitor)) OR (Cytotoxic T Lymphocyte Associated Protein 4 Inhibitor)) OR (CTLA-4 Inhibitor)) OR (CTLA 4 Inhibitor)) OR (PD-1 Inhibitors)) OR (PD 1 Inhibitors)) OR (Programmed Cell Death Protein 1 Inhibitor)) OR (Programmed Cell Death Protein 1 Inhibitors)) OR (PD-1 Inhibitor)) OR (Inhibitor, PD-1)) OR (PD 1 Inhibitor)) OR (Immune Checkpoint Blockade)) OR (Checkpoint Blockade, Immune)) OR (Immune Checkpoint Inhibition)) OR (Checkpoint Inhibition, Immune)) OR (PD-L1 Inhibitors)) OR (PD L1 Inhibitors)) OR (Programmed Death-Ligand 1 Inhibitors)) OR (Programmed Death Ligand 1 Inhibitors)) OR (PD-L1 Inhibitor)) OR (PD L1 Inhibitor)) OR (PD-1-PD-L1 Blockade)) OR (Blockade, PD-1-PD-L1)) OR (PD 1 PD L1 Blockade)) OR (Pembrolizumab)) OR (Nivolumab)) OR (Atezolizumab)) OR (Ipilimumab)) OR (Camrelizumab)) OR (Sintilimab)) OR (Tislelizumab)) OR (Toripalimab)) OR (Envafolimab)) OR (Avelumab)) OR (Tremelimumab)) OR (Durvalumab)) OR (Cemiplimab)))

Web of science(15)

(((Carcinoma, Hepatocellular) OR ((((((((((((((((((Carcinomas, Hepatocellular) OR (Hepatocellular Carcinomas)) OR (Hepatocellular Carcinoma)) OR (Hepatoma)) OR (Hepatomas)) OR (Liver Cancer, Adult)) OR (Adult Liver Cancer)) OR (Adult Liver Cancers)) OR (Cancer, Adult Liver)) OR (Cancers, Adult Liver)) OR (Liver Cancers, Adult)) OR (Liver Cell Carcinoma)) OR (Carcinoma, Liver Cell)) OR (Carcinomas, Liver Cell)) OR (Cell Carcinoma, Liver)) OR (Cell Carcinomas, Liver)) OR (Liver Cell Carcinomas)) OR (Liver Cell Carcinoma, Adult))) AND ((Proton Pump Inhibitors) OR ((((Inhibitors, Proton Pump) OR (Proton Pump Inhibitor)) OR (Inhibitor, Proton Pump)) OR (Pump Inhibitor, Proton)))) AND ((Immune Checkpoint Inhibitors) OR ((((((((((((((((((((((((((((((((((((((((((((((Checkpoint Inhibitors, Immune) OR (Immune Checkpoint Blockers)) OR (Checkpoint Blockers, Immune)) OR (Immune Checkpoint Inhibitor)) OR (Checkpoint Inhibitor, Immune)) OR (CTLA-4 Inhibitors)) OR (CTLA 4 Inhibitors)) OR (Cytotoxic T-Lymphocyte-Associated Protein 4 Inhibitors)) OR (Cytotoxic T Lymphocyte Associated Protein 4 Inhibitors)) OR (Cytotoxic T-Lymphocyte-Associated Protein 4 Inhibitor)) OR (Cytotoxic T Lymphocyte Associated Protein 4 Inhibitor)) OR (CTLA-4 Inhibitor)) OR (CTLA 4 Inhibitor)) OR (PD-1 Inhibitors)) OR (PD 1 Inhibitors)) OR (Programmed Cell Death Protein 1 Inhibitor)) OR (Programmed Cell Death Protein 1 Inhibitors)) OR (PD-1 Inhibitor)) OR (Inhibitor, PD-1)) OR (PD 1 Inhibitor)) OR (Immune Checkpoint Blockade)) OR (Checkpoint Blockade, Immune)) OR (Immune Checkpoint Inhibition)) OR (Checkpoint Inhibition, Immune)) OR (PD-L1 Inhibitors)) OR (PD L1 Inhibitors)) OR (Programmed Death-Ligand 1 Inhibitors)) OR (Programmed Death Ligand 1 Inhibitors)) OR (PD-L1 Inhibitor)) OR (PD L1 Inhibitor)) OR (PD-1-PD-L1 Blockade)) OR (Blockade, PD-1-PD-L1)) OR (PD 1 PD L1 Blockade)) OR (Pembrolizumab)) OR (Nivolumab)) OR (Atezolizumab)) OR (Ipilimumab)) OR (Camrelizumab)) OR (Sintilimab)) OR (Tislelizumab)) OR (Toripalimab)) OR (Envafolimab)) OR (Avelumab)) OR (Tremelimumab)) OR (Durvalumab)) OR (Cemiplimab))) (Topic)

Ovid(Embase)(40)

((Carcinoma, Hepatocellular or (Carcinomas, Hepatocellular or Hepatocellular Carcinomas or Hepatocellular Carcinoma or Hepatoma or Hepatomas or Liver Cancer, Adult or Adult Liver Cancer or Adult Liver Cancers or Cancer, Adult Liver or Cancers, Adult Liver or Liver Cancers, Adult or Liver Cell Carcinoma or Carcinoma, Liver Cell or Carcinomas, Liver Cell or Cell Carcinoma, Liver or Cell Carcinomas, Liver or Liver Cell Carcinomas or Liver Cell Carcinoma, Adult)) and (Proton Pump Inhibitors or (Inhibitors, Proton Pump or Proton Pump Inhibitor or Inhibitor, Proton Pump or Pump Inhibitor, Proton)) and (Immune Checkpoint Inhibitors or (Checkpoint Inhibitors, Immune or Immune Checkpoint Blockers or Checkpoint Blockers, Immune or Immune Checkpoint Inhibitor or Checkpoint Inhibitor, Immune or CTLA-4 Inhibitors or CTLA 4 Inhibitors or Cytotoxic T-Lymphocyte-Associated Protein 4 Inhibitors or Cytotoxic T Lymphocyte Associated Protein 4 Inhibitors or Cytotoxic T-Lymphocyte-Associated Protein 4 Inhibitor or Cytotoxic T Lymphocyte Associated Protein 4 Inhibitor or CTLA-4 Inhibitor or CTLA 4 Inhibitor or PD-1 Inhibitors or PD 1 Inhibitors or Programmed Cell Death Protein 1 Inhibitor or Programmed Cell Death Protein 1 Inhibitors or PD-1 Inhibitor or Inhibitor, PD-1 or PD 1 Inhibitor or Immune Checkpoint Blockade or Checkpoint Blockade, Immune or Immune Checkpoint Inhibition or Checkpoint Inhibition, Immune or PD-L1 Inhibitors or PD L1 Inhibitors or Programmed Death-Ligand 1 Inhibitors or Programmed Death Ligand 1 Inhibitors or PD-L1 Inhibitor or PD L1 Inhibitor or PD-1-PD-L1 Blockade or Blockade, PD-1-PD-L1 or PD 1 PD L1 Blockade or Pembrolizumab or Nivolumab or Atezolizumab or Ipilimumab or Camrelizumab or Sintilimab or Tislelizumab or Toripalimab or Envafolimab or Avelumab or Tremelimumab or Durvalumab or Cemiplimab))).af.


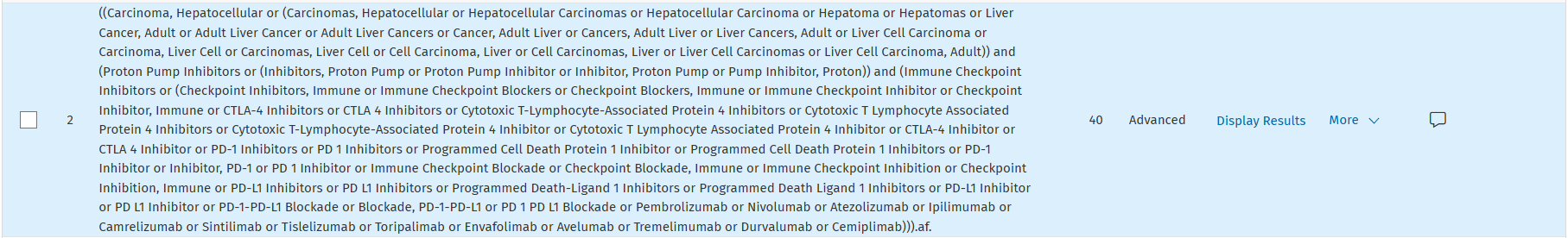

Supplement: Supplementary file 1 [file Table1.docx]
