# Supplementary material for: The influence of concomitant proton pump inhibitors use on treatment efficacy in hepatocellular carcinoma patients receiving immune checkpoint inhibitors: a systematic review and meta-analysis
Source: Front Immunol. 2026 Feb 3;17:1717420. doi: 10.3389/fimmu.2026.1717420 (PMC12960652; doi:10.3389/fimmu.2026.1717420)
Supplement: Supplementary file 2 [file Table2.doc]

| OS | | | |
| --- | --- | --- | --- |
| Study ID | HR | lci | uci |
| Jun 2021 | 1.14 | 0.84 | 1.54 |
| Hatanaka 2023 | 0.96 | 0.66 | 1.42 |
| Wang 2024 | 1.125 | 0.728 | 1.738 |
| Hobeika 2024 | 1.11 | 0.71 | 1.74 |
| Ng 2024 | 0.87 | 0.58 | 1.29 |

| PFS | | | |
| --- | --- | --- | --- |
| Study ID | HR | lci | uci |
| Hatanaka 2023 | 0.96 | 0.66 | 1.42 |
| Hobeika 2024 | 1 | 0.66 | 1.52 |
| Ng 2024 | 0.84 | 0.6 | 1.2 |
